# 2014 ISCB Accomplishment by a Senior Scientist Award: Gene Myers

**DOI:** 10.1371/journal.pcbi.1003621

**Published:** 2014-05-22

**Authors:** Christiana N. Fogg, Diane E. Kovats

**Affiliations:** 1 Freelance Science Writer, Kensington, Maryland, United States of America; 2 Executive Director, International Society for Computational Biology, La Jolla, California, United States of America

The International Society for Computational Biology (ISCB; http://www.iscb.org) annually recognizes a senior scientist for his or her outstanding achievements. The ISCB Accomplishment by a Senior Scientist Award honors a leader in the field of computational biology for his or her significant contributions to the community through research, service, and education. Dr. Eugene “Gene” Myers of the Max Planck Institute of Molecular Cell Biology and Genetics in Dresden has been selected as the 2014 ISCB Accomplishment by a Senior Scientist Award winner.

Myers (Image 1) was selected by the ISCB's awards committee, which is chaired by Dr. Bonnie Berger of the Massachusetts Institute of Technology (MIT). Myers will receive his award and deliver a keynote address at ISCB's 22nd Annual Intelligent Systems for Molecular Biology (ISMB) meeting. This meeting is being held in Boston, Massachusetts, on July 11–15, 2014, at the John B. Hynes Memorial Convention Center (https://www.iscb.org/ismb2014).

**Figure:**
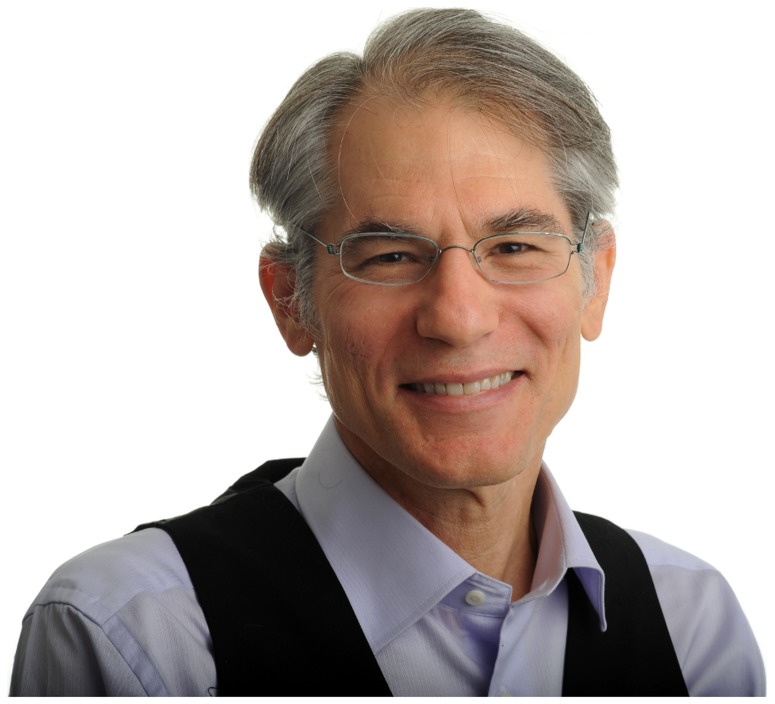
Image 1. Gene Myers. Image credit: Matt Staley, HHMI.

Myers was captivated by computer programming as a young student. He remembered his fascination being stoked by a realization and recalls, “A computer is a programmable device, and once programmed, is a specific device for doing something that I conceived of. I found this magical.” He completed a BS in mathematics at the California Institute of Technology, but his interest in biology came during his PhD studies at the University of Colorado in the late 1970s. Myers recounted that he initially considered molecular biology as “a source of interesting computational questions.” He was studying computer science under the guidance of his dissertation advisor, Andrzej Ehrenfeucht, who had eclectic interests that included molecular biology. Myers, along with fellow graduate students and future bioinformaticians Gary Stormo and David Haussler, was drawn by Ehrenfeucht's curiosity about such basic questions as how to compare DNA sequences and how to build evolutionary trees.

Myers landed his first faculty position in the Department of Computer Science at the University of Arizona. Throughout his research career, he has been interested in sequence assembly. “I first started thinking about this problem in 1984,” he recollected. “While I developed many seminal algorithms for sequence comparison and search in the '80s and early '90s, including BLAST (Basic Local Alignment Search Tool), the problem that has and continues to fascinate me to this day is sequence assembly.” He is well-known for being one of the authors of the 1990 manuscript that first described BLAST, a groundbreaking algorithm that is still used today for sequence comparison. This paper is also one of the most cited papers in scientific literature.

Myers's deep-seated interest in sequence assembly led him to promote the idea that whole genome shotgun sequencing, which had been effective in sequencing microbial genomes, could be used on the large and unwieldy human genome. Craig Venter believed in this approach as well, and he brought Myers to Celera Genomics in 1998 during their push to sequence the human genome. Myers recalled writing thousands of lines of code to build algorithms that could assemble the vast amounts of sequence data. He considers the success of this landmark sequencing project as a highlight of his career.

In 2002, Myers returned to academia in a position at the University of California, Berkeley's Center for Integrative Genomics. Several animal genomes had been sequenced, and many more were in progress. Myers and others were getting to work on mining the mounting collection of genetic information and developing algorithms to compare genomes at an unprecedented scale.

More recently, Myers headed a lab at the Howard Hughes Medical Institute (HHMI)'s Janelia Farm Research Campus, and in 2012, he moved to Dresden, Germany, and became director at the Max Planck Institute of Molecular Cell Biology and Genetics and the Klaus-Tschira chair of the Systems Biology Center. Myers describes that his “latest focus all started because [he] wish[es], like many, to ‘decode’ the genome.” His work has evolved into building microscopic devices and image analysis tools that can be used to observe and model the inner workings of cells and biological systems. “Having the genome of an organism allows one to engage in transgenics on a systematic, genome-wide scale,” he explained. “In a way, we can now ‘stain’ or instrument any genomic entity of interest. What better way to understand function than to watch what individual elements of the genome do? I am particularly interested in how collections of hundreds of thousands of cells orchestrate and coordinate their function to produce tissues of a given function and shape. Genomes encode this; how?”

Myers acknowledged that microscopy and computational bioimaging are hindered by limits of image resolution, the often weak signals produced by imaging reagents, and the inability to robotically control commercial microscopes. Myers and his research group are working to overcome some of these barriers by building their own microscopic devices. He sees this type of work as having the potential to revolutionize medicine. He said, “Really understanding (in molecular terms) what a cell can do and how what it does affects its role in a complex tissue or organ will greatly advance medicine and treatment as well as help us understand variation across species and how organisms develop.”

Myers recounted the importance of key mentors in the success of his career. Myers met Webb Miller when he was a young faculty member at the University of Arizona in the early 1980s. The two struck up a fruitful collaboration that led to many early papers about sequence analysis. Myers explained, “Miller helped me greatly in the early part of my career in that he taught me, through example, that writing can be fun.” Myers gained a different sort of insight while working for Venter at Celera. He described Venter as “a master of the sound bite, and while this may sound trivial, it is actually more important than one might think. Much of one's career success depends on the ability to present one's ideas in powerful, succinct, clear ways.”

Myers believes that mentorship should be “about shaping the character of the individual and their understanding of their role within the research community.” He starts with himself as he aims to “do [his] best to be a good role model, to instill values of integrity, objectivity, and openness.” He states, “I see my role as mentor as simply to create a vibrant intellectual atmosphere that allows my students to blossom.” Myers has trained students from varied academic backgrounds throughout his career, and he considers that passion as well as talent are vital to the success of his trainees. Myers affirmed that “there is no substitute for passion.”

Myers has also benefitted by working in diverse research climates. He explained that “universities or private research institutions are the best places for basic research.” He also sees a place for biotech and startups, like Celera. “If you want to actually do something big,” he said, “then a startup (if it makes financial sense) is a much, much faster way to get it done.” Despite the pros and cons associated with both of these settings, Myers feels that “private universities or not-for-profit institutions, such as the Max-Planck-Gesselschaft (MPG) that I belong to now, are terrific in that there is more flexibility of funding, and thus, it is easier to take risks and work towards long-term visions.”

Myers's unique contributions to computational biology have been recognized by several awards, including election to the National Academy of Engineering (2003), the Association for Computing Machinery (ACM) Kannellakis Prize (2002), and the International Max Planck Research Prize (2004). Bonnie Berger (MIT), chair of the ISCB Nominating Committee, sees Myers as an exemplar of the Achievement by a Senior Scientist Award. Berger stated that, “Myers is one of the founders of the field, bringing his algorithmic expertise to the most fundamental problems in computational biology. From his role in creating the indispensable and widely used BLAST program for basic sequence search, to breaking the barrier of sequencing the human genome, to deciphering what is coded in DNA, he has launched our discipline. Myers has been a prominent member of the ISCB community, serving on the Board of Directors, as an ISCB fellow, and as chair and area chair for numerous ISMB meetings.” Alfonso Valencia, leader of the Structural Computational Biology group at the Spanish National Cancer Research Center and president-elect of ISCB, also sees Myers as a stellar representative of the field. Valencia said of this year's award winner, “I am particularly happy about the election of Gene Myers, since he represents the strong roots of computational biology in algorithmic and method development. The intensity with which he lives science, the originality of his approaches, and the attention he dedicates to the technical details are characteristics of his work and a great example for our new generations of bioinformaticians and computational biologists.”

Myers remains fervent and passionate about the work he does. He contends that his upbringing, which included traveling the globe with his family, as well as his innate passion for science and mathematics have helped make him “flexible, broad-minded, and curious.” He also prefers to keep his research group small while keeping his research vision large. He advises future and active scientists, “Simultaneously be able to ‘go deep’ and yet continuously remain in an environment that keeps you in touch with the ‘big picture.’ And you always have to take on new challenges and new problems.” In the end, he cannot speak strongly enough about the importance of passion and states, “My overarching advice is to do what you are passionate about. Ours is not a career for security or wealth. You have to love it, absolutely love it.”

